# Supplementary material for: The effects of GLP-1 receptor agonists on visceral fat and liver ectopic fat in an adult population with or without diabetes and nonalcoholic fatty liver disease: A systematic review and meta-analysis
Source: PLoS One. 2023 Aug 24;18(8):e0289616. doi: 10.1371/journal.pone.0289616 (PMC10449217; doi:10.1371/journal.pone.0289616)

Fig. S1 Sensitivity Analysis Chart comparing the posttreatment Visceral Fat of the control and GLP-1RA groups.


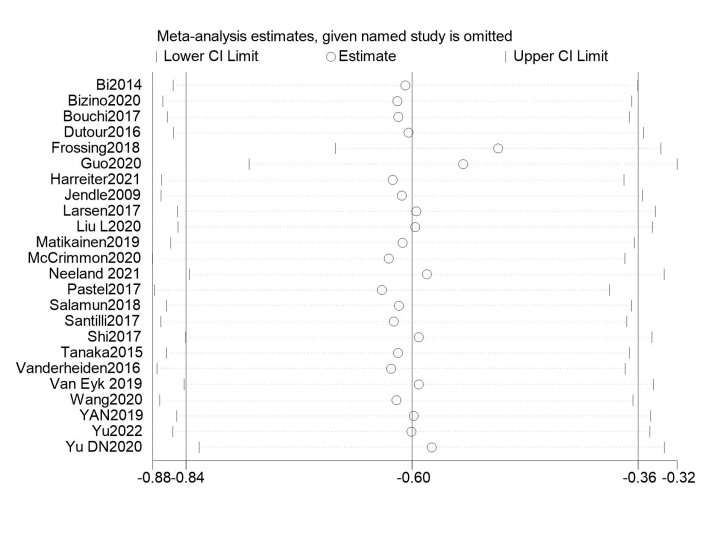


Fig. S2 Sensitivity Analysis Chart comparing the posttreatment Hepatic Fat Content of the control and GLP-1RA groups.


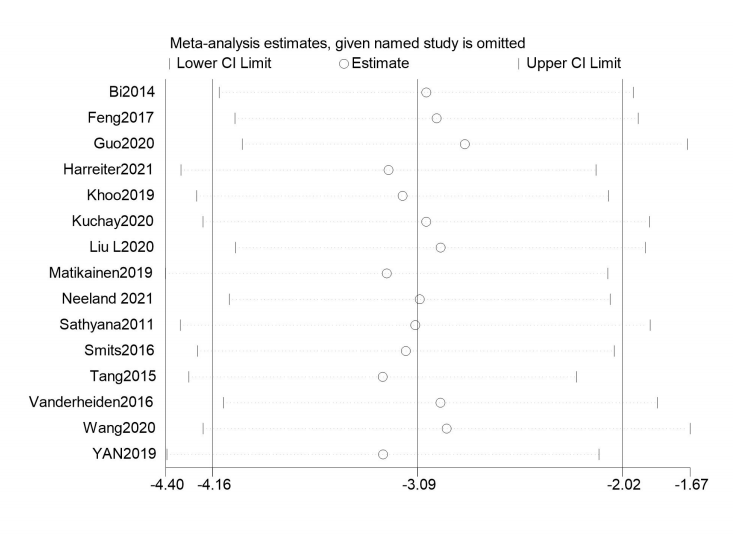


Fig. S3 Sensitivity analysis chart of visceral fat in Type 2 diabetes control group and GLP-1RA group after treatment.


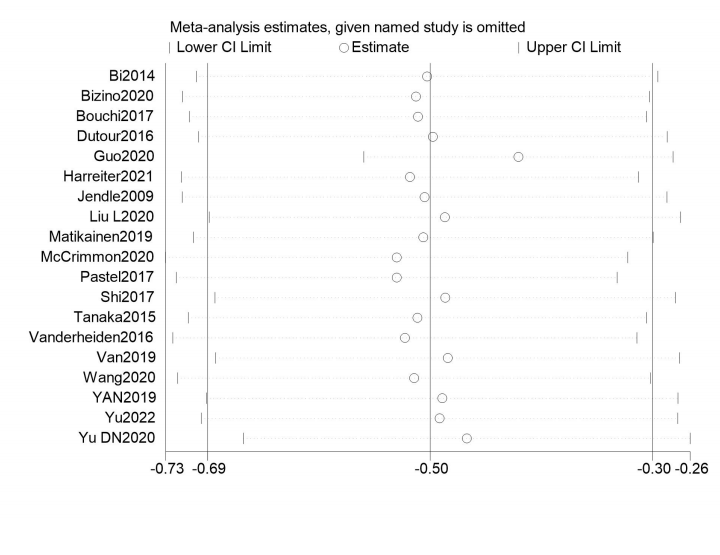


Fig. S4 Sensitivity analysis chart of visceral fat in Non-Type 2 diabetes control group and GLP-1RA group after treatment.


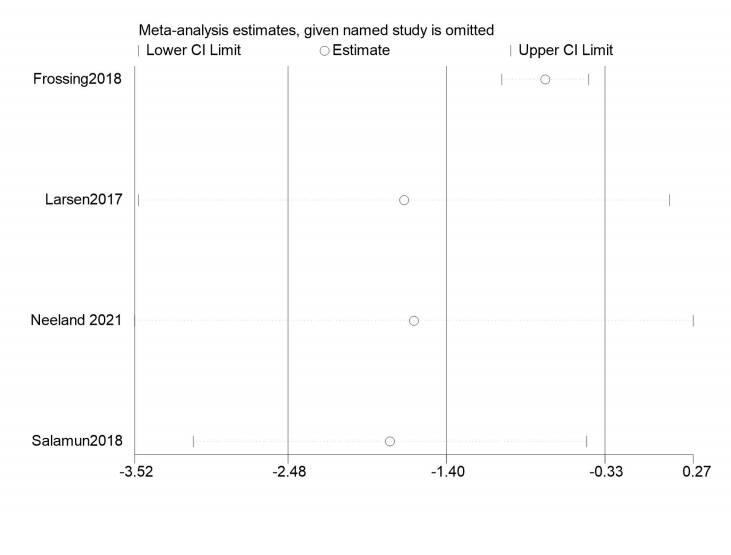


Fig. S5 Sensitivity analysis chart of visceral fat in NAFLD control group and GLP-1RA group after treatment.


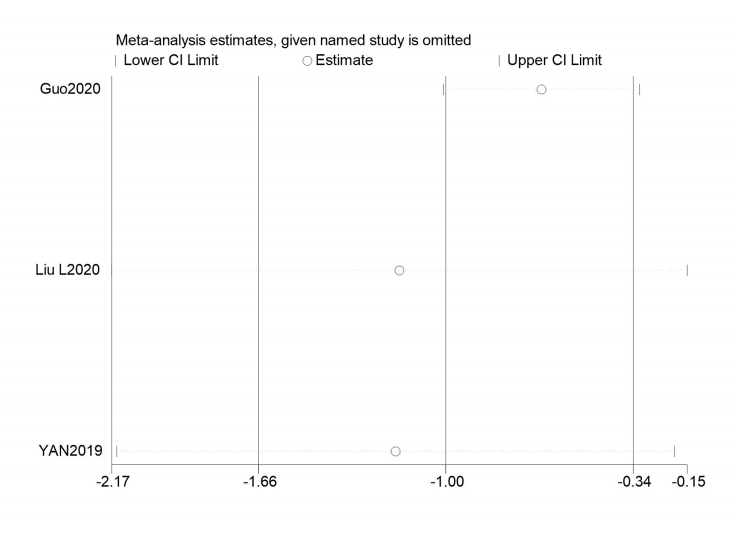


Fig. S6 Sensitivity analysis chart of visceral fat in Non-NAFLD control group and GLP-1RA group after treatment.


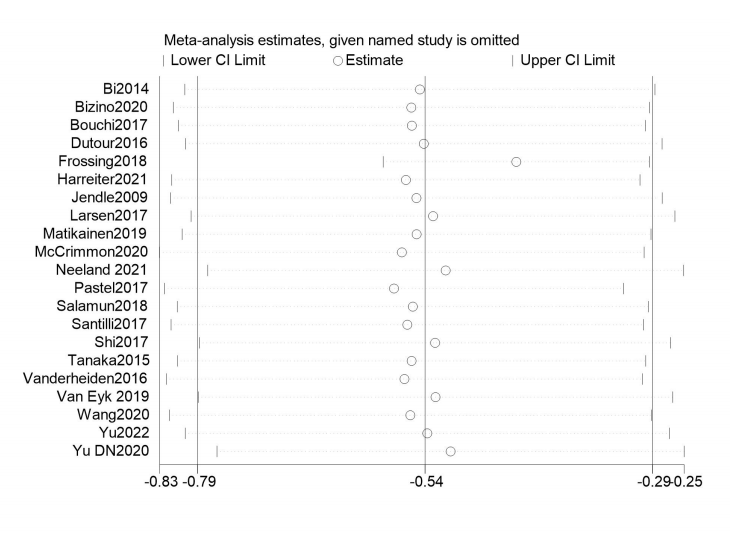


Fig. S7 Sensitivity analysis chart of Hepatic Fat Content in Type 2 diabetes control group and GLP-1RA group after treatment.


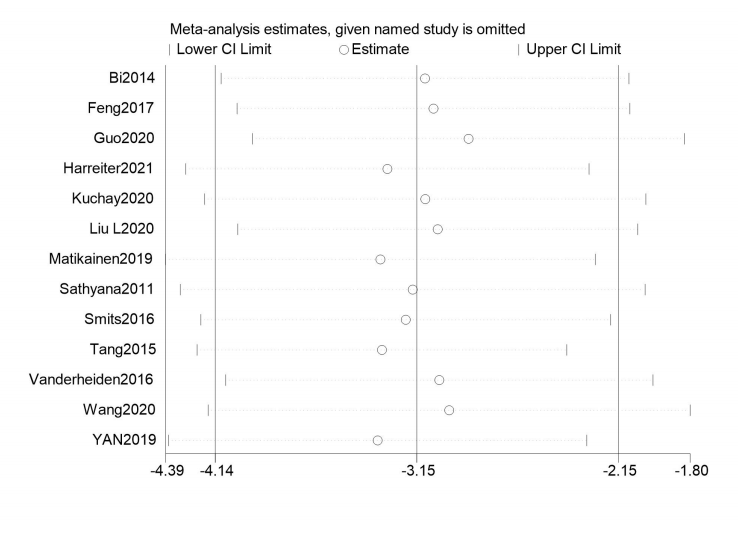


Fig. S8 Sensitivity analysis chart of Hepatic Fat Content in Non-Type 2 diabetes control group and GLP-1RA group after treatment.


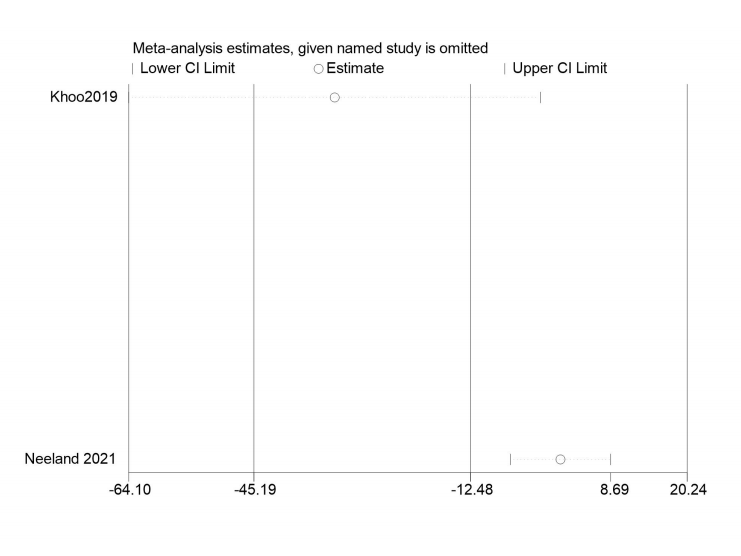


Fig. S9 Sensitivity analysis chart of Hepatic Fat Content in NAFLD control group and GLP-1RA group after treatment.


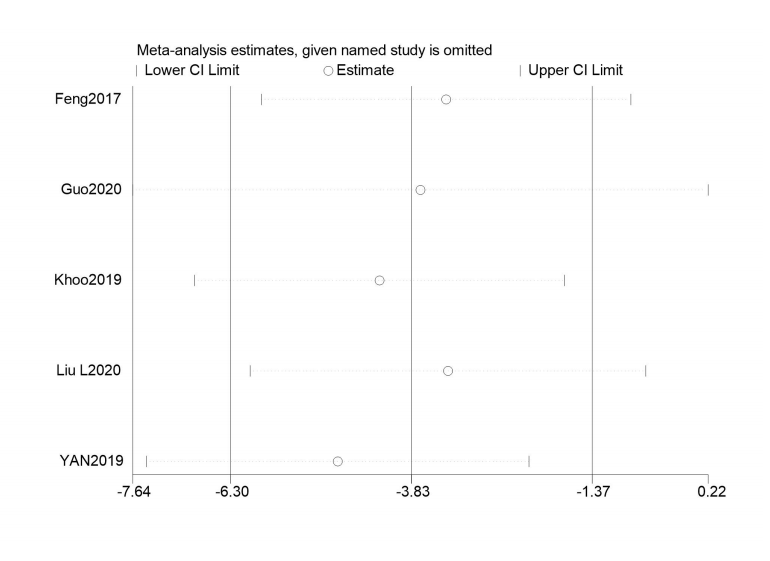


Fig. S10 Sensitivity analysis chart of Hepatic Fat Content in Non-NAFLD control group and GLP-1RA group after treatment.


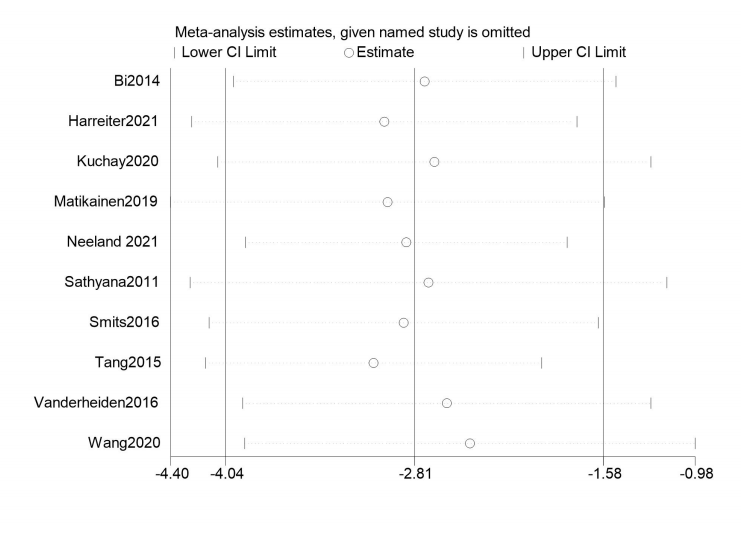


Fig. S10 Sensitivity analysis chart of Hepatic Fat Content in Type 2 diabetes and NAFLD control group and GLP-1RA group after treatment.


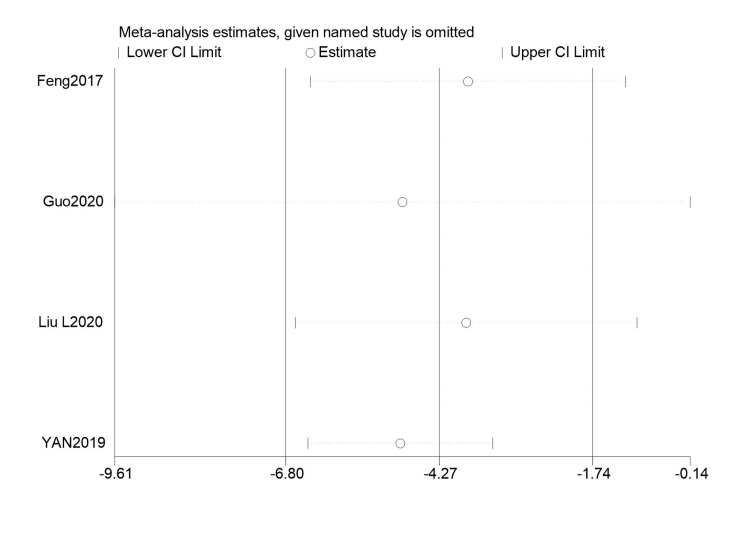


Fig. S11 Forest plot of the effect of GLP-1Ras on visceral fat in subgroups of follow-up time.


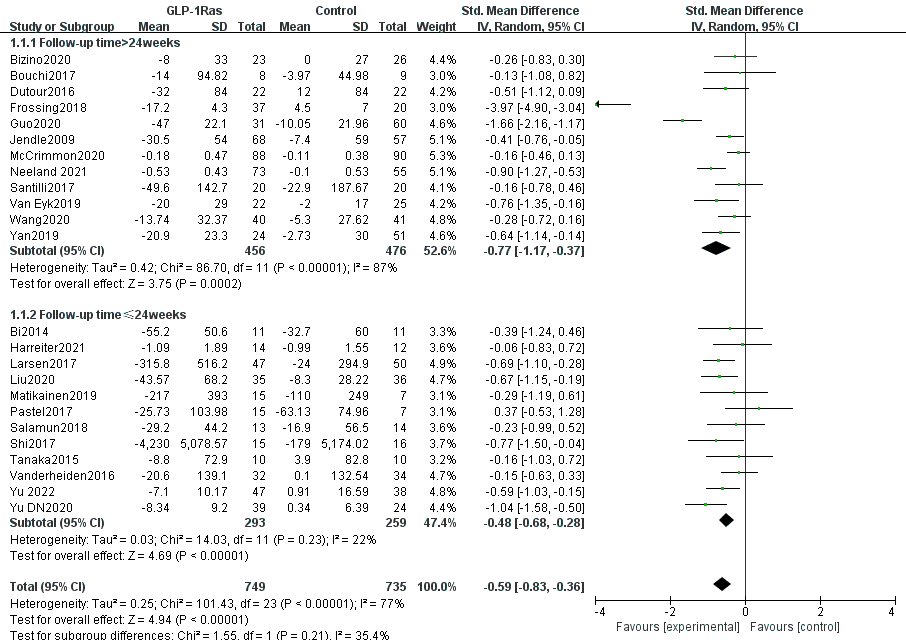


Fig. S12 Forest plot of the effect of GLP-1Ras on visceral fat in subgroups of GLP-1Ras types.


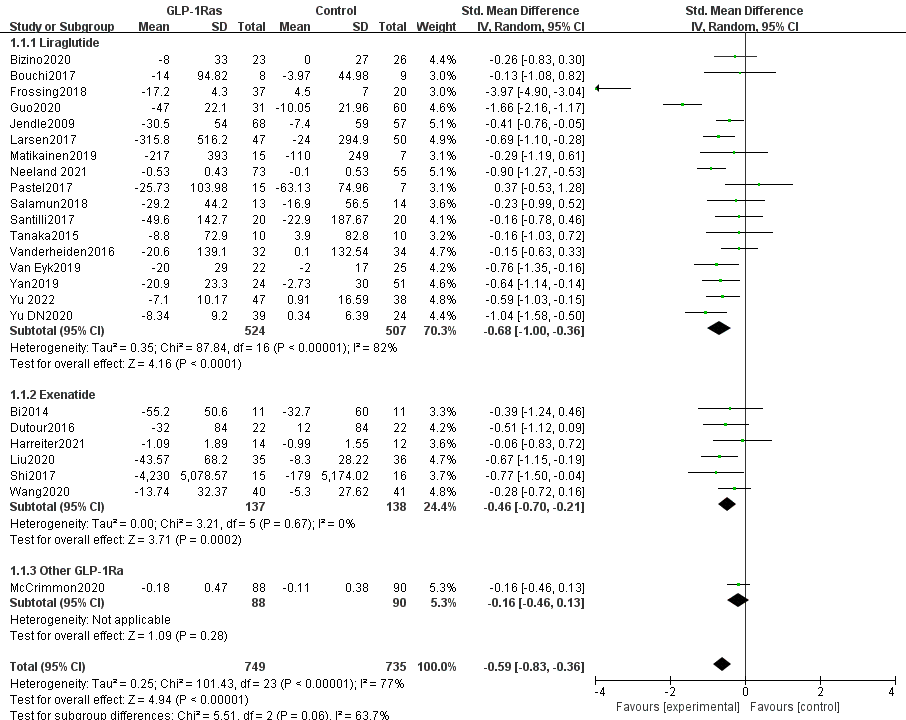


Fig. S13 Forest plot of the effect of GLP-1Ras on visceral fat in subgroups of baseline BMI.


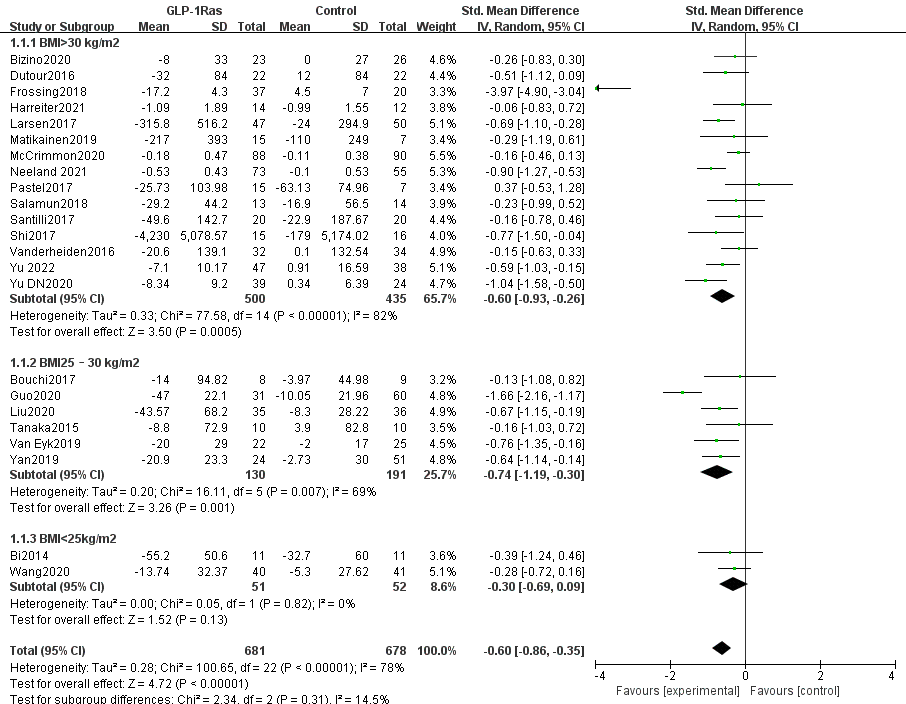


Fig. S14 Forest plot of the effect of GLP-1Ras on visceral fat in subgroups of Age.


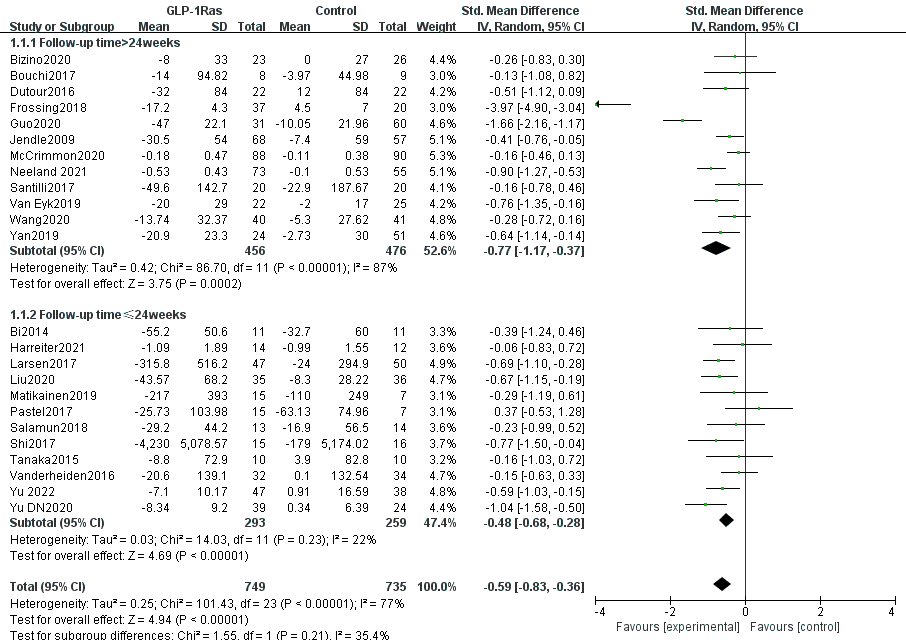


Fig. S15 Forest plot of the effect of GLP-1Ras on visceral fat in subgroups of outcome measures method.


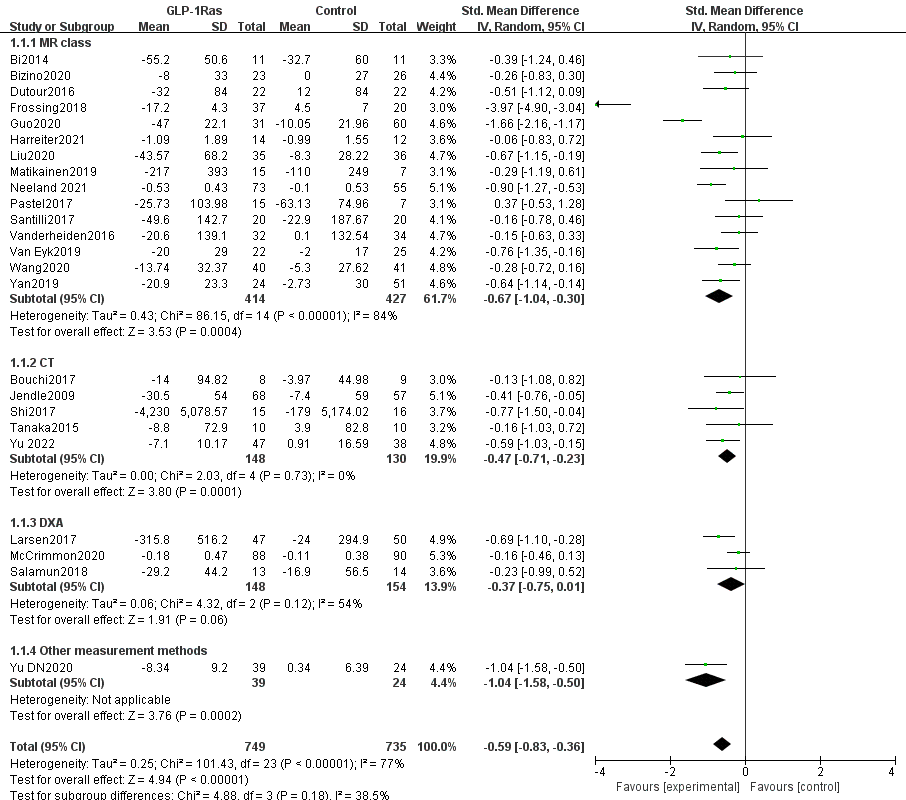


Fig. S16 Forest plot of the effect of GLP-1Ras on hepatic fat content in subgroups of follow-up time.


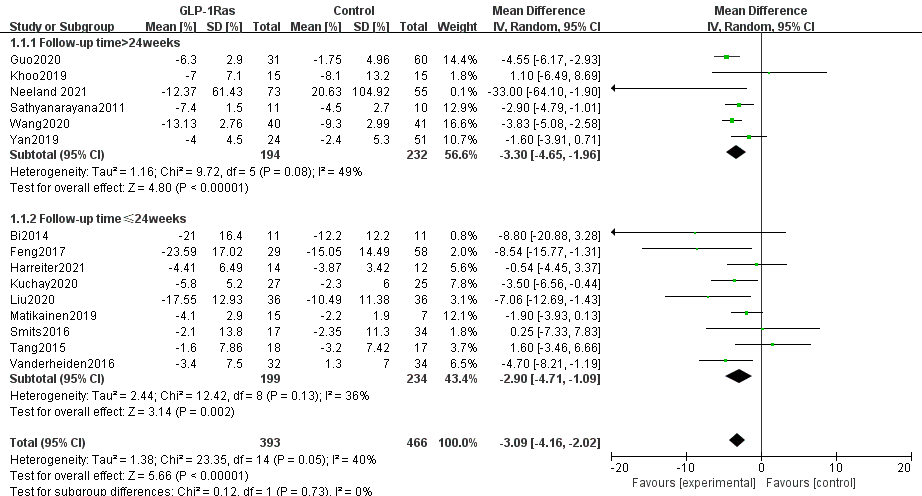


Fig. S17 Forest plot of the effect of GLP-1Ras on hepatic fat content in subgroups of GLP-1Ras types.


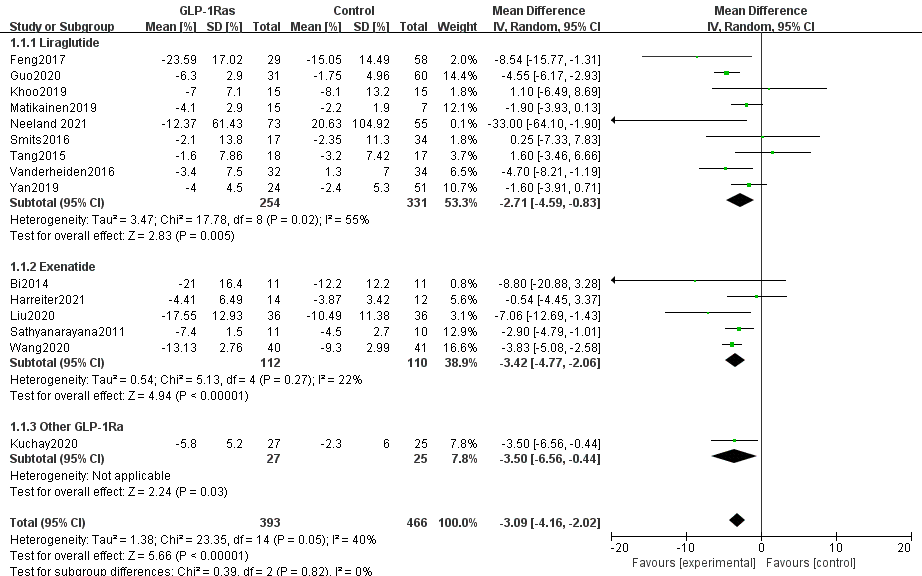


Fig. S18 Forest plot of the effect of GLP-1Ras on hepatic fat content in subgroups of baseline BMI.


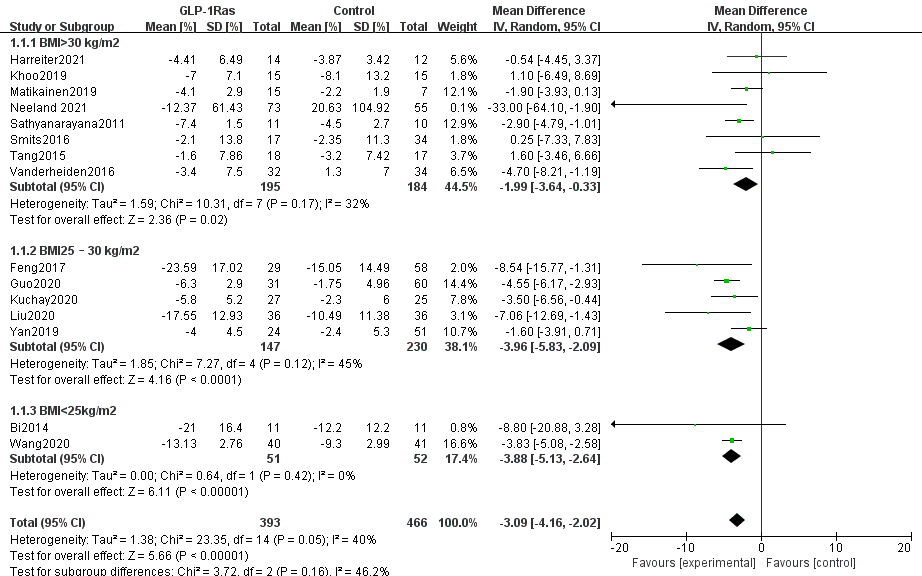


Fig. S19 Forest plot of the effect of GLP-1Ras on hepatic fat content in subgroups of Age.


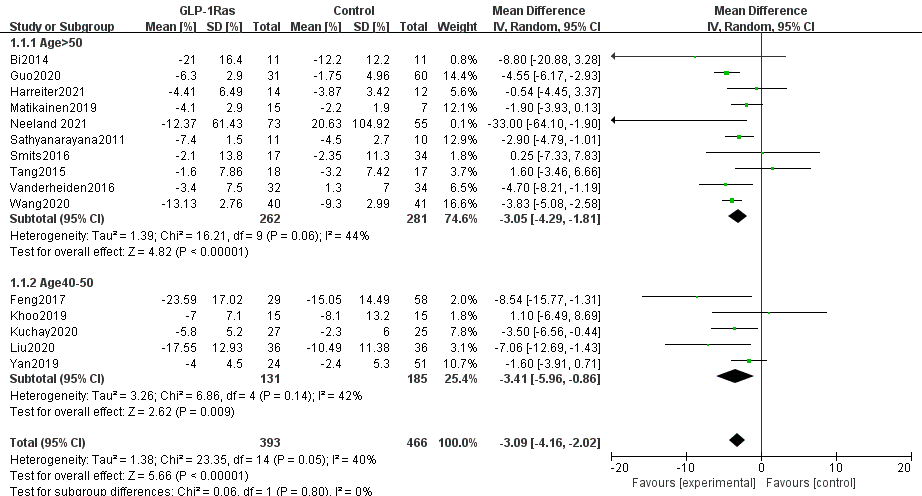


Fig. S20 Forest plot of the effect of GLP-1Ras on hepatic fat content in subgroups of outcome measures method.


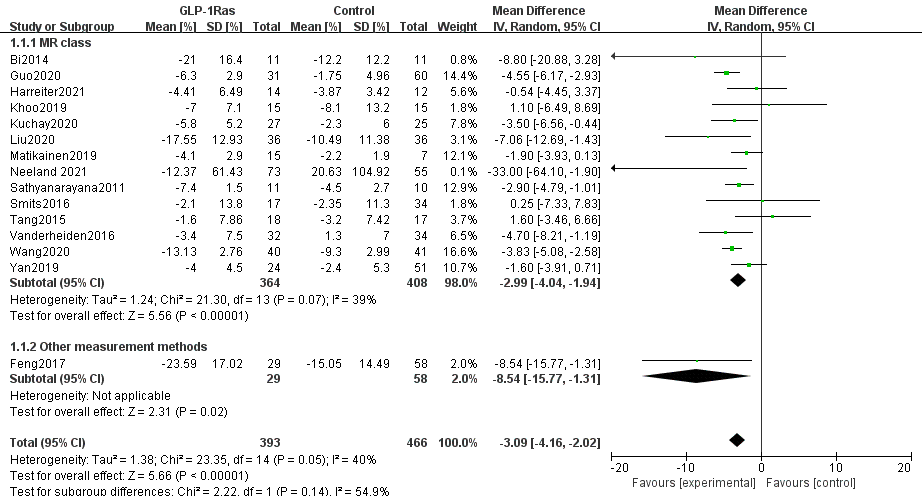


Fig. S21 Meta-regression analysis of the effect of GLP-1Ras type on visceral fat.


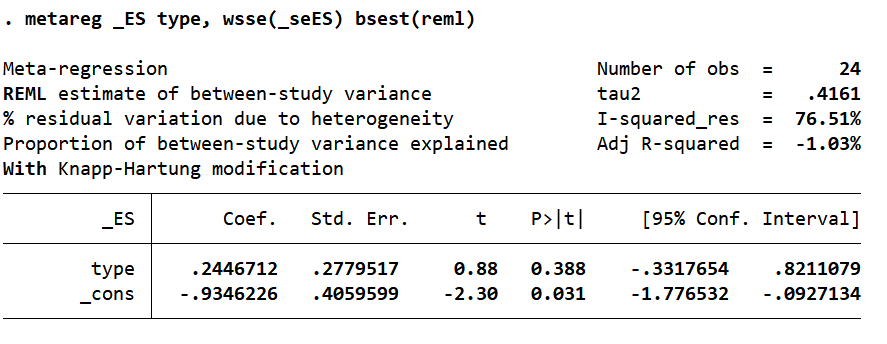


Fig. S22 Meta-regression analysis of the effect of outcome measures method on hepatic fat content.


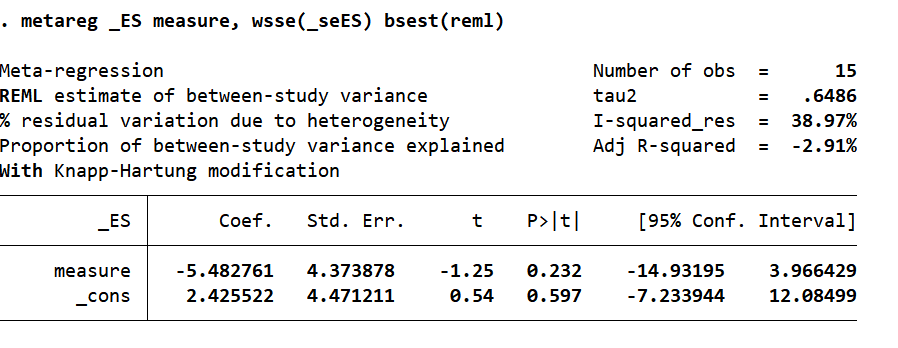

Supplement: S1 File — (DOCX) [file pone.0289616.s002.docx]
