# Supplementary material for: The effects of GLP-1 receptor agonists on visceral fat and liver ectopic fat in an adult population with or without diabetes and nonalcoholic fatty liver disease: A systematic review and meta-analysis
Source: PLoS One. 2023 Aug 24;18(8):e0289616. doi: 10.1371/journal.pone.0289616 (PMC10449217; doi:10.1371/journal.pone.0289616)
Supplement: Attachment — Submitted filename: response letter.docx [file pone.0289616.s003.docx]

Journal Requirements:

When submitting your revision, we need you to address these additional requirements.

1. Please ensure that your manuscript meets PLOS ONE's style requirements, including those for file naming. The PLOS ONE style templates can be found at https://journals.plos.org/plosone/s/file?id=wjVg/PLOSOne_formatting_sample_main_body.pdf and 
https://journals.plos.org/plosone/s/file?id=ba62/PLOSOne_formatting_sample_title_authors_affiliations.pdf

2. Please note that PLOS ONE has specific guidelines on code sharing for submissions in which author-generated code underpins the findings in the manuscript. In these cases, all author-generated code must be made available without restrictions upon publication of the work. Please review our guidelines at https://journals.plos.org/plosone/s/materials-and-software-sharing#loc-sharing-code and ensure that your code is shared in a way that follows best practice and facilitates reproducibility and reuse.

[Note: HTML markup is below. Please do not edit.]

Reviewers' comments:

Reviewer's Responses to Questions

**Comments to the Author**

1. Is the manuscript technically sound, and do the data support the conclusions?

The manuscript must describe a technically sound piece of scientific research with data that supports the conclusions. Experiments must have been conducted rigorously, with appropriate controls, replication, and sample sizes. The conclusions must be drawn appropriately based on the data presented.

Reviewer #1: Yes

2. Has the statistical analysis been performed appropriately and rigorously?

Reviewer #1: Yes

3. Have the authors made all data underlying the findings in their manuscript fully available?

The [PLOS Data policy](http://www.plosone.org/static/policies.action#sharing) requires authors to make all data underlying the findings described in their manuscript fully available without restriction, with rare exception (please refer to the Data Availability Statement in the manuscript PDF file). The data should be provided as part of the manuscript or its supporting information, or deposited to a public repository. For example, in addition to summary statistics, the data points behind means, medians and variance measures should be available. If there are restrictions on publicly sharing data—e.g. participant privacy or use of data from a third party—those must be specified.

Reviewer #1: Yes

4. Is the manuscript presented in an intelligible fashion and written in standard English?

PLOS ONE does not copyedit accepted manuscripts, so the language in submitted articles must be clear, correct, and unambiguous. Any typographical or grammatical errors should be corrected at revision, so please note any specific errors here.

Reviewer #1: Yes

5. Review Comments to the Author

Please use the space provided to explain your answers to the questions above. You may also include additional comments for the author, including concerns about dual publication, research ethics, or publication ethics. (Please upload your review as an attachment if it exceeds 20,000 characters)

Reviewer #1: Liao et al has conducted a meta-analysis on “The effects of GLP-1 receptor agonists on visceral fat and liver ectopic fat in an adult population with or without diabetes and nonalcoholic fatty liver disease” and concluded that e GLP-1 receptor agonists significantly reduce visceral and liver fat content in adults. These findings were especially true for patients with both type 2 diabetes and NAFLD, hence suggest that GLP-1Ras should be more widely used in the treatment of these conditions. This can prove to be valuable finding, however, in my opinion, the manuscript can be improved by incorporating the following edits:
1. The abstract exceeds the word limit of 250 words and is very lengthy. The authors should consider summarizing the content and providing only the main findings of the study in the abstract.

Response: Thank you for your helpful comments. We shortened the abstract accordingly.

2. In introduction, the authors should use expanded form of NAFLD it was used here the first time in the manuscript other than the abstract.

Response: Thank you for your helpful comments. We expanded the NAFLD in the first use in the introduction.

3. In 2nd paragraph of the ‘introduction’, the authors should write the abbreviation of GLP-1 receptor agonists as ‘GLP-1 RAs’. Moreover, the authors have used ‘GLP-1 Ras’ as the abbreviation elsewhere in the manuscript, which should be corrected.

Response: Thank you for your critical comments. We revised it accordingly.

4. In the ‘data extraction and quality assessment’ section, the authors should explain all the outcomes, primary and secondary, and specify subgroups for better comprehensibility of the manuscript.

Response: Thank you for your helpful comments. We detailed the primary and secondary outcomes and specified the subgroups.

5. The numbering of the sections of the results is not correct. The authors should correct the numbering of the subheadings to avoid confusion in the readers.

Response: Thank you for your critical comments. We remumbered the section of the results.

6. In subsection ‘Visceral fat in different population groups’ of the results, the authors have stated that “Of the 24 studies reporting the effect of GLP-1Ras on visceral fat, 19 studies had participants with type 2 diabetes, three with NAFLD, four without type 2 diabetes, and 21 with type 2 non-NAFLD.” The authors should correct the part ‘type 2 non-NAFLD’ by removing ‘type 2’ to make better sense of the information.

Response: Thank you for your helpful comments. We revised it accordingly.

7. For all outcomes in the ‘results’, provide a reference for the forest plot showing the result of that outcome.

Response: Thank you for your helpful comments. We provided a reference for each forest plot in the result section.

8. The authors should make a comparison of this meta-analysis with previously existing studies to highlight the significance of this study and how it adds to the current literature.

Response: Thank you for your critical comments. We compared our work with previously existing studies.

9. 2nd, 3rd and 4th paragraphs of the discussion provide a very detailed content of the number of conditions predisposed by obesity. However, this information is not in relevance with the scope of this study in as much detail. This information could be summarized into few lines and linked to clinical implications of the findings of this study. This would help maintain relevance and coherence.

Response: Thank you for your insightful comments. We rewrote these detailed content of the number of conditions predisposed by obesity accordingly.

10. The authors should consider providing a distinct paragraph in the discussion for ‘clinical implications’ to highlight the significance of the findings in clinical practice.

Response: Thank you for your insightful comments. We added the clinical implications in the discussion.

6. PLOS authors have the option to publish the peer review history of their article ([what does this mean?](https://journals.plos.org/plosone/s/editorial-and-peer-review-process#loc-peer-review-history)). If published, this will include your full peer review and any attached files.


If you choose “no”, your identity will remain anonymous but your review may still be made public.


**Do you want your identity to be public for this peer review?** For information about this choice, including consent withdrawal, please see our [Privacy Policy](https://www.plos.org/privacy-policy).

Reviewer #1: No
